# Milk Analysis and Infant Feeding

**Published:** 1886-01

**Authors:** 


MILK ANALYSIS AND INFANT FEEDING. A Practical Treatise on the Examination of Human and Cows' Milk, Cream, Condensed Milk, etc., and Directions as to the Diet of Young Infants. Arthur V. Meigs, M. D., Physician to the Pennsylvania Hospital and to the Children's Hospital; Fellow of the College of Physicians of Philadelphia, etc. Philadelphia: P. Blakiston, Son & Co., No. 1012 Walnut street. 1885.

Much that is contained in this work has already been published in the Transactions of the Philadelphia Medical Society and the College of Physicians of Philadelphia. It appears now in a handsome little book of 102 pages. The work is well written, and deserves to be in the hands of every physician.

